# The value of the visual evoked potentials test in the assessment of the visual pathway in head trauma

**DOI:** 10.5249/jivr.v13i1.1525

**Published:** 2021-01

**Authors:** Pejvak Azadi, Morteza Movassat, Mohammad Hosein Khosravi

**Affiliations:** ^*a*^ Eye Research Center, Emam khomeini Hospital, Kermanshah University of Medical Sciences, Kermanshah, Iran.; ^*b*^ Eye Research Center, Farabi Eye Hospital, Tehran University of Medical Sciences, Tehran, Iran.

**Keywords:** Visual evoked potential, VEP, Visual pathways, Head trauma, Head injury Neuroimaging

## Abstract

**Background:**

The research was done to evaluate the value of the visual evoked potentials test in the assessment of visual pathways function in cases with head trauma and minimal findings on routine testing.

**Methods:**

A prospective case series evaluating use of visual evoked potentials testing in patients with a history of head trauma and suffering from visual symptoms with no significant clinical and neuroimaging findings, referred for further work up.

**Results:**

Thirty-four patients with a history of head trauma and subsequent visual complaints were included. 27 cases (79.4%) were male and 7 cases (20.6%) were female. The mean elapsed time after the trauma was 47.6 weeks (range: 3.5 to 320 weeks). Twenty-five cases had unilateral and 9 cases had bilateral visual complaints. History of coma with mean duration of 12 days was present in 4 cases. The best-corrected visual acuity was less than 1 Log MAR (legally blind) in 21 eyes. In 4 eyes (12%) the relative afferent papillary defect test was positive. Mild to moderate optic disc pallor was present bilaterally in 4 cases and unilaterally in 3 cases. Hemorrhagic patches were reported on MRI in 2 cases; no other cases had pathologic MRI findings. In unilateral cases, there was a statistically significant difference between the involved eye-sided lobe and the sound eye-sided lobe implicit time and amplitude. In patients with bilateral complaints, by testing each eye, the VEP amplitudes of both eyes showed significant differences with the International Society for Clinical Electrophysiology of Vision standards, whereas the implicit times showed not-statistically significant differences.

**Conclusions:**

The visual evoked potentials test shows not only additional diagnostic value, not seen on routine clinical and neuroimaging testing, but also rather a high validity in tracing visual disability in traumatic brain injury.

## Introduction

Signs and symptoms of visual disturbances, which may appear early or late after the trauma, could present a diagnostic challenge for clinicians. The reason depends not only on the complexity of the central nervous system but also on the need to differentiate between true symptoms and malingering or factitious disorders. The first and most effective step is a thorough clinical examination to show the physical and psychological condition of the patient and also to predict the final visual outcomes. 

In cases with head injury and ophthalmic complaints, neuroimagings especially CT scan and MRI are usually necessary to rule out abnormalities in visual pathways. Van Stavern et al. reported a study on 326 consecutive patients with head trauma, mostly due to motor vehicle accidents. ^1^ They found abnormal neuro-ophthalmic findings in 56.7% of their cases,^1^ and reported that both afferent and efferent pathways are vulnerable to trauma, especially the optic nerve which may be damaged directly or indirectly in concussions. Although they believe that visual field (VF) testing can help to find the location of the lesion, they also note that VF testing is poorly reliable, especially in the acute phase of trauma. D.A. Jacobs and S.L. Galetta emphasized combining neuroimaging with proper ophthalmic examinations in these cases, ^2^ but even more sophisticated neuroimaging techniques, like functional MRI could not trace all trauma induced brain lesions; as Bruce lee and Andrew Newberg claimed, in a high percentage of cases with minor head trauma, the CT scan or MRI reveal nothing abnormal. ^3^ They also emphasized that white matter changes could be found in many healthy middle-aged individuals, and may not be indicative of post-traumatic changes. These considerations made many experts to think about other methods to evaluate the visual pathways in brain traumas, especially those objective and functional tests such as visual evoked potentials (VEP) which measures the light stimulus responses as electrical signals over the occipital lobe scalp. This test could be performed in uncooperative patients like children and cases in especial physical condition.

In agreement with these works, we studied the VEP changes in 34 patients with post head trauma visual complaints and unreasonable clinical and neuroimaging findings.

## Materials and Methods

From January to December 2018, patients with visual complaints after head trauma, who were referred to the electrodiagnostic clinic of the Farabi eye hospital, were included in this case series. The patients were referred from either neuro-ophthalmogists because of a discrepancy between the severity of their complaints and the clinical and neuroimaging findings, or from forensic medicine centers to prove their financial claims. Full ophthalmic examinations including best-corrected visual acuity (BCVA), relative afferent pupillary defect (RAPD), extraocular muscle function, tonometry, slit lamp examination and funduscopy were performed in all cases. In patients with good fixation ability (23 cases), pattern/reversal VEP with 30 check size, 50 cd/m^2^ illumination and 98 % contrast, while asking the patient to sit 100 cm away from the monitor was performed. In cases with poor fixation ability (11 cases) flash VEP with 60 Hz frequency and 100 cd/m^2^ illumination and having the patient sat 33 cm away from the monitor was performed. Using the ISCEV 2009 updated guidelines, ^4^ each eye was tested separately while the contralateral eye was occluded with dark pads. The electrodiagnosis unit was MonPack 3 from Metrovision Company, France. The Aliguille EEG needles and for temporal skull skin the ELE12 electrodes were used. Statistical analyses were performed using SPSS for Windows version 18.0 (SPSS, Chicago, IL, United States).

## Results

Thirty-four cases with a history of trauma and subsequent reported visual disturbances who were referred to the electrodiagnostic clinic of the Farabi eye hospital included in the analysis. 25 cases had unilateral and 9 cases had bilateral complaints of decreased vision. 27 cases (79.4%) were male and 7 cases (20.6%) were female. The mean elapsed time after the trauma was 47.6 weeks (range: 3.5 to 320 weeks). BCVA was less than 1 Log MAR (legally blind) in 21 eyes. In 4 eyes (12%) the RAPD was positive and mild to moderate optic disc pallor was present bilaterally in 4 cases and unilaterally in 3 cases. History of coma with mean duration of 12 days was present in 4 cases. Hemorrhagic patches were reported in 2 cases in MRI, in which the location and size of the patches could not describe the visual complaints, in other cases no pathologic findings were found in MRI. As shown in Table 1, in unilateral cases, by using the paired t-test, the involved-side-lobe measurements showed a statistically significant longer implicit time (P value<0.000) and lower amplitudes (for the right lobe and the left lobe the P values were 0.05 and 0.007, respectively) than the sound-side-lobe measurements. In bilateral cases, the measurements were compared with ISCEV standards (Table 1, Table 2 and 3). As shown in Table 3, the measured amplitudes of both eyes for the both lobes were statistically significant less than the ISCEV standards (P values<0.001). The implicit times of the both eyes for the both lobes were longer than the ISCEV standards although not statistically significant difference was found. (P values>0.05).

**Table 1:** Paired t-test comparing VEP results in 25 cases with unilateral complaints.

VEP recordings	Sound side	Involved side	P value
Right lobe implicit time(ms)	110.288 ± 12.02	131.080 ± 20.48	0.000
Left lobe implicit time(ms)	108.584 ± 10.76	127.849 ± 19.61	0.000
Right lobe Amplitude(mV)	9.872 ± 5.14	8.208 ± 6.39	0.05
Left lobe Amplitude(mV)	11.496 ± 6.05	8.864 ± 7.50	0.007

**Table 2:** Normal values in pattern VEP and flash VEP according to the ISCEV standards.

	Implicit time P2 (ms)	Amplitude P2 (µV)
flash VEP	Average= 112, SD=11	Average=9, SD=5
	Implicit time P100 (ms)	Amplitude P100 (µV)
Pattern30’	Average=106, SD=3	Average=16, SD=7

**Table 3:** T-test comparing VEP results in 9 cases with bilateral complaints, with ISCEV standard values.

VEP recordings	Mean ± SD	Difference with ISCEV standards	P value
First eye right lobe implicit time	116.889 ± 23.63	10.88	0.204
First eye left lobe implicit time	115.600 ± 23.620	9.60	0.250
Second eye right lobe implicit time	118.00 ± 22.880	12.00	0.150
Second eye left lobe implicit time	118.11 ± 19.483	12.111	0.099
First eye right lobe amplitude	6.189 ± 3.580	-9.8111	0.000
First eye left lobe amplitude	6.333 ± 6.0809	-9.666	0.001
Second eye right lobe amplitude	4.711 ± 4.0790	-11.288	0.000
Second eye left lobe amplitude	5.40 ± 5.9795	-10.600	0.001

## Discussion

As many neurologists and neuro-ophthalmologists, we find the evaluation of the post head trauma visual disability challenging. Considering the nature of trauma related brain lesions, especially in mild injuries with no gross anatomical changes, this evaluation is even more complicated. Although clinical examinations, including visual acuity and RAPD, and neuroimaging are the first and most important diagnostic steps, in cases of microscopic tissue damages with no obvious MRI findings, and also in evaluation of the cases suspicious for malingering, we need an objective functional test.^5^ Perimetry is another invaluable test in any case of CNS lesions, but it is neither valid in legal cases who may have financial claims, nor operable in unconscious and uncooperative patients, and others have mentioned the limitations of these tests in MTB injuries.^6,7^ When trauma causes lesions in optic nerve head and retinal nerve fiber layer (RNFL), ocular coherence tomography (OCT), may have a role to document the injury but as RT Naismith and colleagues mentioned, this method could be only considered as a complementary test to VEP.^6^

In these situations VEP seems to be valid as an objective functional test, not only performable with general anesthesia in cases with extraordinary conditions, but also having diagnostic and prognostic values.^7^ On the other hand, the post trauma vision syndrome (PTVS) includes not only visual symptoms, but also sensory motor dysfunctions like diplopia, vertigo and even hallucination-like experiences. By performing VEP in PTVS cases and comparing its results to the results of normal cases, Wv Padula et al. found this test as a reliable method to evaluate the cortical binocular integrate function.^8^ Near to this, is the study of Keneth J Ciuffred et al. who emphasize on visual motion sensitivity changes in mild traumatic brain (MTB) injury.^9^ They performed VEP test with binasal occlusionin both normal cases and cases with brain trauma, and showed an increase in the VEP amplitude in traumatic cases. As they said, there is a habitual attempt in head trauma cases to suppress the visual information in the retinal periphery to reduce the abnormal motor sensitivity. Emphasizing the value of VEP in post trauma brain injury, Yadav NK and Ciuffred KJ claimed that changes of the checker size and contrast can affect the VEP results.^10^ By using a 20’ checker and low contrast stimulation, they found a correlation between the time elapsed after the trauma and VEP amplitudes. Jihoon Jeon and co-workers suggested pattern VEP for visual acuity quantification in disabled patients and to evaluate any probable simulation. They believe that amplitudes more than 5.77 microvolts in pattern VEP could show a good visual performance.^11^

Our study included 34 cases of head injury, complaining of visual symptoms, with either no positive clinical or neuroimaging findings in many of them, or no reasonable proportion between the severity of symptoms in few cases and the positive clinical or neuroimaging findings. As our results show VEP can trace CNS lesions causing visual disability even in cases with no obvious clinical and paraclinical findings. When VEP has been shown previously as an efficacious test in assessing non-traumatic CNS lesions and anomalies, this study could show its value more.^12-14^ For example, Pojd-Wilczek reported VEP results in ocular involvement due to systemic disorders;^15^ JelkaBrecelj et al. showed the VEP results in 2 cases of achiasmatic children, ^16^ and John P. Kelly showed the reliability of VEP in the optic nerve glioma.^17^ Utility of VEP test will be more emphasized by giving attention to CS Hoyt’s report who mentioned about a high proportion of the brain volume which subserves the vision, and concluded that the visual pathways are largely prone to head traumas. ^18^ Due to these reasons, it seems necessary to have a test with high sensitivity and specificity for both diagnosis and prognosis in these cases. We are in full agreement with Feinsod and Auerbach who draw attention to the importance of clinical features in monitoring the progress of the traumatic brain dysfunction, ^19^ but we would like to emphasize severely on more reliable objective and functional techniques like VEP, with capability to perform in unconscious or uncooperative patients.

Although our study shows the importance of electrodiagnostic tests measured by VEP in trauma-related brain dysfunction, further studies with a larger sample size and longer follow up period are suggested.

## Conclusion

Visual evoked potentials test, especially pattern/ reversal VEP, is a useful objective and functional test to evaluate the visual pathways function in CNS disorders due to head trauma.
